# LSD1: an emerging face in altering the tumor microenvironment and enhancing immune checkpoint therapy

**DOI:** 10.1186/s12929-023-00952-0

**Published:** 2023-07-31

**Authors:** M A A Mamun, Yu Zhang, Jin-Yuan Zhao, Dan-Dan Shen, Ting Guo, Yi-Chao Zheng, Li-Juan Zhao, Hong-Min Liu

**Affiliations:** 1 Key Laboratory of Advanced Drug Preparation Technologies, Ministry of Education, China, State Key Laboratory of Esophageal Cancer Prevention and Treatment, Key Laboratory of Henan Province for Drug Quality and Evaluation, Institute of Drug Discovery and Development, School of Pharmaceutical Sciences, Zhengzhou University, 100 Kexue Avenue, Zhengzhou, 450001 China; 2 Department of Obstetrics and Gynecology, The Third Affiliated Hospital of Zhengzhou University, Zhengzhou, 450052 Henan China; 3 Key Laboratory of Endometrial Disease Prevention and Treatment Zhengzhou China, The Third Affiliated Hospital of Zhengzhou University, Zhengzhou, 450052 Henan China; 4 State Key Laboratory of Esophageal Cancer Prevention and Treatment, Academy of Medical Sciences, Zhengzhou University, Zhengzhou, 450052 China

**Keywords:** LSD1, Tumor microenvironment, Immunosuppression, Immune checkpoint blockade, Epigenetic TME regulation

## Abstract

Dysregulation of various cells in the tumor microenvironment (TME) causes immunosuppressive functions and aggressive tumor growth. In combination with immune checkpoint blockade (ICB), epigenetic modification-targeted drugs are emerging as attractive cancer treatments. Lysine-specific demethylase 1 (LSD1) is a protein that modifies histone and non-histone proteins and is known to influence a wide variety of physiological processes. The dysfunction of LSD1 contributes to poor prognosis, poor patient survival, drug resistance, immunosuppression, etc., making it a potential epigenetic target for cancer therapy. This review examines how LSD1 modulates different cell behavior in TME and emphasizes the potential use of LSD1 inhibitors in combination with ICB therapy for future cancer research studies.

## Introduction

LSD1, also known as KDM1A, AOF2, and BHC110, is one of the key histone demethylases involved in the epigenetic regulation of gene expression [1, 2]. This is the initial discovery of a histone demethylase responsible for eliminating mono- and dimethyl modifications from histones 3 lysine 4 (H3K4), histone 3 lysine 9 (H3K9), and histone 4 lysine 20 (H4K20). Consequently, it regulates both transcriptional repression and activation [3]. To date, various functions of LSD1 have been reported such as enzymatic actions on histone and non-histone proteins [4], scaffolding functions [5], and part of multiprotein complexes [6]. In its initial discovery, LSD1 was found to act as a transcriptional repressor by removing methyl groups from the active mono- and di-methylated histone 3 lysine 4 marks (H3K4me1, H3K4me2) [7]. To fulfill this role, LSD1 interacts with a REST corepressor (Co-REST), forming a complex [8, 9]. Subsequent studies have indicated that LSD1 demethylates monomethylated lysine 9 (H3K9me1) and dimethylated lysine 9 (H3K9me2) on histone H3, resulting in transcriptional activation [10]. However, this activity is regulated in an androgen receptor (AR) dependent manner. Upon complexing with AR, LSD1 demethylates the repressive H3K9 and thereby promotes gene activation [11]. The increasing interest in drug targets for LSD1 stems from its frequent overexpression and commonly observed negative correlation with prognosis in numerous types of cancer. Currently, multiple LSD1 inhibitors are undergoing clinical trials and demonstrate considerable potential in the field of cancer treatment [12]. Epigenetic alterations, such as DNA methylation, histone modification, and noncoding RNA regulation, contribute to the diverse expression patterns of human genes [13]. Besides, numerous published studies consistently demonstrate that epigenetic alterations have a significant impact on the reprogramming of the TME [14]. The TME consists of various cellular components, including immune cells, stromal cells, and extracellular matrix, along with soluble factors and signaling molecules. Multiple types of cancer are influenced by the TME [15]. It plays a crucial role in both immune activation and suppression within the context of cancer. Besides, the TME employs several mechanisms to suppress immune responses. One such mechanism involves the upregulation of immune checkpoints, such as programmed death-1 (PD-1)/programmed death-ligand 1 (PD-L1), cluster of differentiation 86 (CD86)/cytotoxic T-lymphocyte antigen 4 (CTLA-4) [16]. These checkpoints act as molecular brakes on immune cells, preventing excessive activation and potential damage to healthy tissues. However, cancer cells exploit these checkpoints to evade immune surveillance and suppress antitumor immune responses. Consequently, the field of cancer treatment has undergone a revolutionary transformation with the advent of ICB therapy. This innovative approach enhances the patient's immune system to target and eliminate tumor cells. The remarkable achievements witnessed in various cancers using monoclonal antibodies (mAb) targeting CTLA-4 and PD-1, exemplify the effectiveness and power of ICB therapy strategies [17, 18]. There are still significant barriers to therapeutic success because of tumor-specific antigens (TA) and toxicities associated with treatment [19]. However, emerging evidence from multiple studies suggests that combining ICB with the targeting of epigenetic marks can be an effective strategy in cancer treatment [20, 21]. DNA methyltransferase inhibitors and histone deacetylase inhibitors have been utilized to enhance the antitumor immune response [22]. Epigenetic therapy can sensitize cancer cells to immune attack, leading to the targeted destruction of tumor cells while sparing normal cells [23]. This targeted approach can minimize off-target toxicities often associated with traditional chemotherapy or radiotherapy. Interestingly, LSD1 has also emerged as a key regulator during the migration and functioning of various immune cells, including T cells, macrophages, natural killer cells (NK), myeloid-derived suppressor cells (MDSCs), and dendritic cells (DCs), within the TME. Recent studies have shed light on the significant role of LSD1 in orchestrating immune cell dynamics within the TME, and its potential impact on improving the therapeutic efficacy of ICB therapies, such as anti-PD-L1 or anti-PD-1 therapies [24, 25]. A deeper understanding of the functions of LSD1 within the TME can provide valuable insights into the mechanisms of ICB resistance and open new avenues for combining LSD1 inhibitors with immunotherapy in cancer treatment.

### Structural skeleton and featured functions of LSD1

**Fig. 1:**
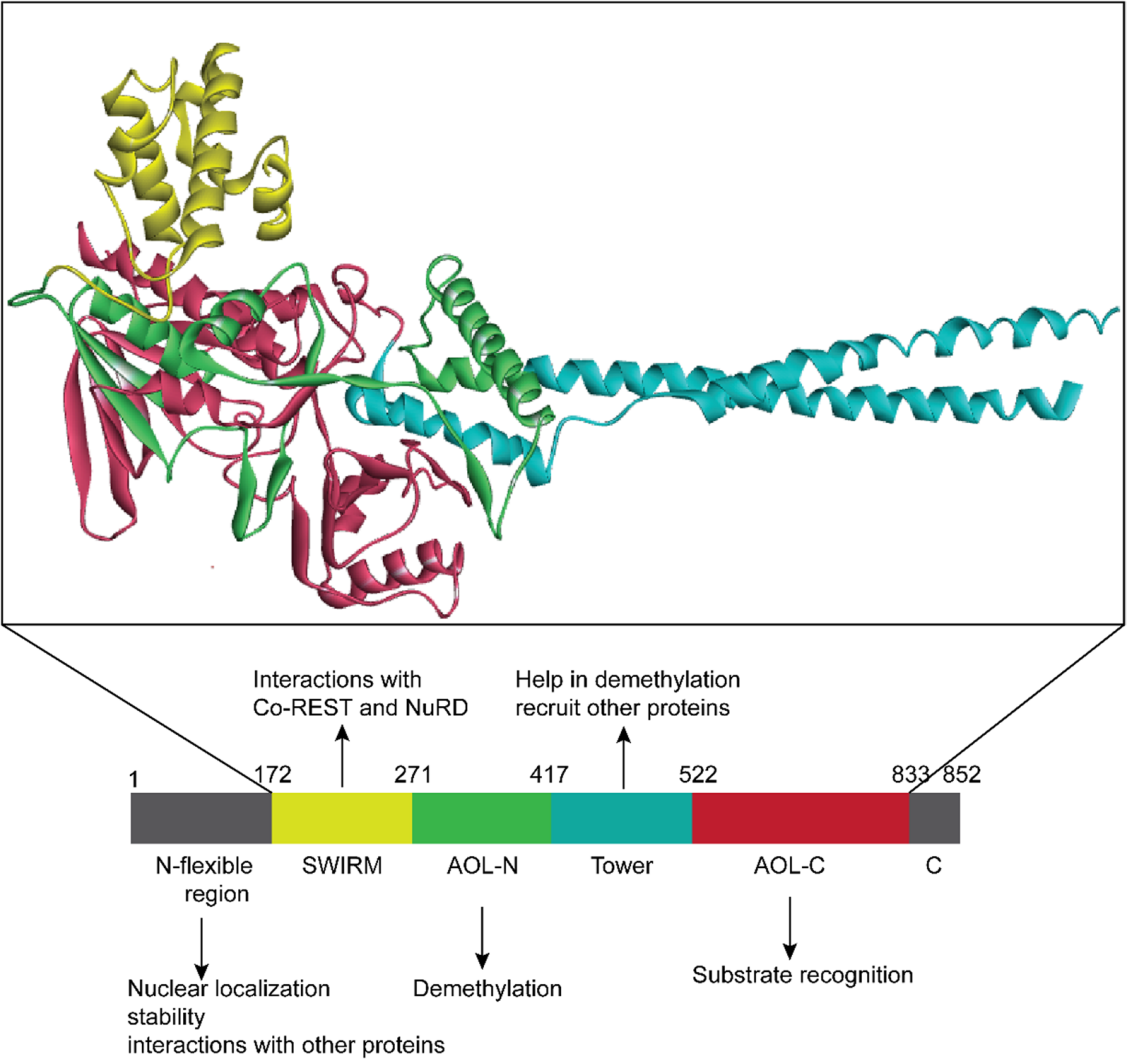
Structural features of the various parts of LSD1. The crystal structure of the protein was obtained from RCSB PDB (ID:2HKO). N-flexible region and SWIRM domain mostly function as interactions with other proteins, whilst AOL-N and TOWER domain operate demethylase activity. The AOL-C domain recognizes specific substrates to demethylase. N-flexible region and C domain have not shown in the crystal structure

LSD1 is a highly conserved flavin-dependent monoamine oxidase (MAO) protein that spans 852 amino acids (aa) in length and has a molecular weight of 110 kDa. The protein is characterized by three major domains, each serving distinct functions and contributing to its structural organization (Fig. 1) [26]. Despite not being classified as a major domain, the N-flexible region of LSD1 (aa 1–171) plays a crucial role in nuclear localization, protein interactions, and post-translational modifications (PTMs) [9, 27]. The sequence indicates that following the N-terminus, the SWI3/RCS8/MOIRA (SWIRM) domain spans amino acids 172 to 271. This domain consists of α-helices that fold onto the catalytic C-terminus of the protein. Several proteins involved in chromatin regulation and modulation contain SWIRM domains [28]. Unlike conventional SWIRM domains that bind to DNA, the SWIRM domain found in LSD1 plays a different role by enhancing its stability and acting as a docking site for interacting with various proteins like nucleosome remodeling deacetylase (NuRD) and CoREST [6]. The second domain is an amine oxidase-like domain (AOL) that regulates LSD1’s enzymatic activity, targeting substrate proteins through its catalytic center. This domain is composed of two lobes. The first lobe (aa 271–416) is located at the N-terminal site and structurally binds with the SWIRM domain, which contains flavin adenine dinucleotide (FAD) binding sites and contributes to oxidation. The catalytic residue, Lys661, engages in an interaction with the buried FAD molecule located within the deepest hydrophobic region of the pocket [29]. A mutation at Lys661 of LSD1 renders it incapable of functioning as a demethylase [30]. The second lobe (522–832 aa) is located at the C-terminus and is responsible for substrate recognition. It plays a vital role in LSD1's ability to bind to additional residues surrounding the target lysine. This includes the first 20 amino acids of histone 3. As a result, LSD1 can effectively accommodate and interact with extended basic histone tails. This structural feature implies that, in addition to its catalytic functions, LSD1 may also specifically bind to a variety of proteins, thereby enabling it to perform non-catalytic functions. For example, research has found that AOL and CoREST domains have an affinity for extra nucleosomal DNA [31]. The third domain, the TOWER domain, is situated between the AOL-N domain and the AOL-C domain. It’s positioning between these domains suggests a role in facilitating communication and coordination between the functional regions of the protein [32]. It protrudes from LSD1's catalytic center and does not appear to make any contact with the rest of the protein. Generally, it consists of an antiparallel coil with two long helices, exhibiting a repetitive pattern of seven residues. This domain is indispensable for the histone demethylase activity of LSD1. In addition, besides its demethylase activity, this domain also contributes to the recruitment of other proteins to LSD1 [32].

Despite the long-standing belief that LSD1 is only responsible for the mono- and di- demethylation of Histone 3 lysine 4 with CoREST, resulting in gene repression, later studies have shown that its demethylation activity on histone H3K9 and H3K20 leads to gene reactivation [7]. However, LSD1 substrates were not limited to only histone proteins. Some non-histone proteins, including E2F transcription factor 1 (E2F1), DNA methyltransferase 1 (DNMT1), tumor suppressor p53, signal transducer and activator of transcription 3 (STAT3), and hypoxia-inducible factor-1 (HIF-1α), have also been included in the list of targets for LSD1 demethylation [33]. In addition to its demethylation activity, LSD1 also serves various biological functions through its scaffolding functions. On one hand, it plays a protective role in preventing the proteasome-dependent degradation of estrogen-related receptor alpha (ERRα), on the other hand, it promotes the proteasomal degradation of F-Box and WD Repeat Domain Containing 7 (FBXW7) [34, 35]. Furthermore, the interaction between LSD1 and autophagy-related protein p62 leads to the stabilization of p62 [36]. Finally, the collaborative efforts of LSD1 and Zinc Finger Protein 217 (ZNF217) synergistically activate the gene network within prostate cancer cells. Importantly, this activation is independent of the demethylase activity of LSD1 or the androgen receptor (AR)-dependent survival pathway in these cancer cells [10].

Recent studies have provided insights into the role of LSD1 in regulating tumor immunity. It has been discovered that the genetic inactivation of LSD1 leads to the downregulation of cluster of differentiation 47 (CD47) and PD-L1 expression by upregulating H3K4me2 levels in their promoter regions. It suggests that LSD1-mediated demethylation of H3K4 is capable of directly regulating the expression of CD47 and PD-L1 [37]. Moreover, significant changes in H3K4 modifications at the PD-1 gene locus were observed when CD8 T cells were stimulated either ex vivo or in vivo. Studies have demonstrated that the absence of LSD1 in stimulated CD8 T cells leads to increased expression of PD-1 mRNA and surface PD-1 [38]. Additionally, numerous studies have reported that LSD1 plays a role in modulating various immune cells and tumor microenvironments [39–41]. We will delve into this topic in detail in the following discussion.

## Role of LSD1 in tumor immune microenvironment regulation

### Effects on various cell type in TIME

**Fig. 2:**
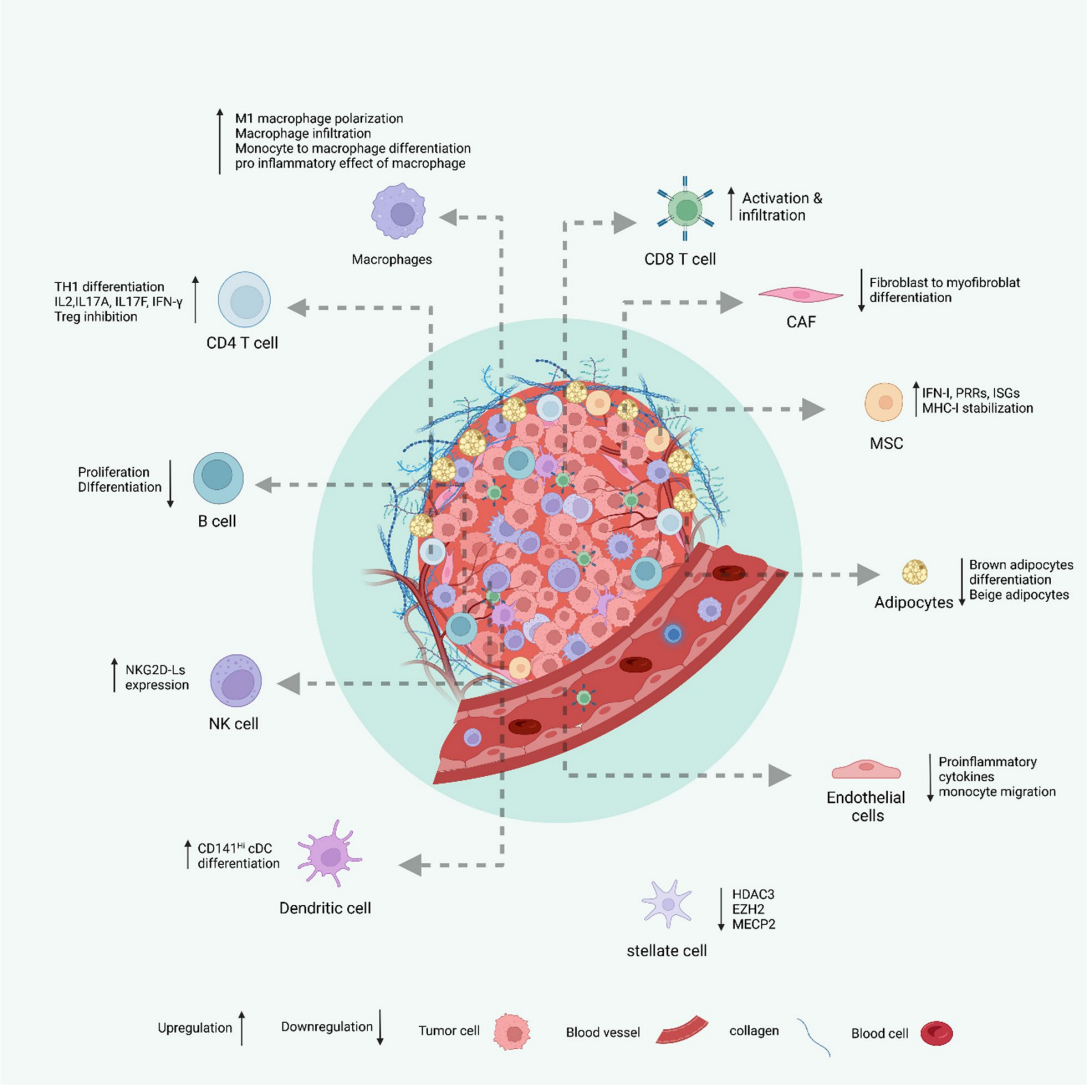
Effect of LSD1 inhibition on various cells in TME. The image demonstrates the impact of inhibiting LSD1 on an assortment of immune and stromal cells within the TME. This process enhances the tumor-eliminating capabilities of CD8 T cells and NK cells, while simultaneously reducing the functionality of immunosuppressive cells. “Figure created with BioRender.com”

Recently, there have been exciting new studies focusing on the impact of LSD1 on different immune cells and stromal cells in TIME. The results have revealed some fascinating findings. Overall, inhibiting LSD1 has been shown to boost the immune activity within the TIME, while also preventing the formation of an immune suppressive environment. Figure 2 provides a visual representation of these studies, and the following section elaborates on the specific details and implications of these observations.

### Immune cell regulation

#### Regulation of T cells

T cells’ epigenetic modifications have been extensively studied in the last decade due to advances in sequencing technologies. Various signals induce extensive epigenomic remodeling in T cells, influencing phenotypic stability and lymphocyte function [42]. By regulating several types of genes, LSD1 also regulates the function of T cells. In humans, LSD1 expression is inversely correlated with CD8 T cell infiltration, as observed in the cancer genome atlas (TCGA) data analysis [43]. One of the best predictors of the immune response for eliminating cancer cells is the number and phenotype of CD8 T cells recruited to the tumor site. It appears that LSD1 inhibition accelerates the tumor infiltration of T cells, as observed in a mathematical model analyzing adaptive immune responses to tumor growth [44]. There is a significant increase in CD4 and CD8 T cell numbers in LSD1 knock-out (KO) compared to control tumors, indicating a stronger ability to induce T cell immunity [24]. However, the cytotoxic activity of CD8 T cells was not significantly changed. These results suggest that a major effect of LSD1 ablation is to promote CD8 T cell infiltration into tumors [24]. There has also been extensive research on the relationship between LSD1 and CD4 T cells. Pharmacologically inhibiting or knocking down LSD1 induces Th1 cell differentiation in activated CD4 T cells. The underlying mechanism involves growth factor independent 1 transcriptional repressor (GFI-1) facilitating the recruitment of LSD1 to specific gene regions associated with T helper type 1 (Th1) cells, including T-box transcription factor protein (TBX21), eomesodermin (EOMES), and runt-related transcription factor 2 (RUNX2). This recruitment leads to a decrease in methylation levels, ultimately resulting in the suppression of interferon-gamma (IFN-γ) production by Th1 CD4 T cells [45]. Nevertheless, the use of small interfering RNA (siRNA) mediated knockdown or small molecule inhibitors targeting LSD1 has revealed that the stimulation of the T-cell receptor (TCR) enhances the production of IFN-γ protein and mRNA in naive CD4 T cells. Furthermore, studies have provided evidence that the interaction between GFI-1 and the intergenic regions of interleukin 17 (IL17) A/F prompts the recruitment of LSD1, resulting in the suppression of IL17A and IL17F expression in TH17-type CD4 T cells [46]. In contrast, the application of a small molecule inhibitor targeting LSD1 liberates the chromatin from both GFI-1 and LSD1. Regulatory T cells (Tregs), another subset of CD4 T cells, play a role in suppressing host antitumor immunity, thereby limiting the effectiveness of tumor immune surveillance. It has been discovered that there exists a physical association between LSD1 and the Treg marker forkhead box P3 (FOXP3). Pharmacological inhibition of LSD1 leads to the production of IL2, IFN-γ, impairs Treg function and enhances antitumor immunity [47]. The adoptive transfer of T cells genetically engineered to express chimeric antigen receptors (CAR) has shown remarkable efficacy in treating various hematological malignancies, including B-cell lymphoma, leukemia, and multiple myeloma. Notably, CAR-T cells targeting the CD19 antigen have produced remarkable clinical outcomes for cancer patients. A study described that anti-CD19 CAR-T cells with downregulated LSD1 demonstrated increased anti-tumor functions both in vitro and in vivo [48]. Overall, the inactivation of LSD1 has been observed to promote the antitumor effects of pro-inflammatory CD8 and CD4 T cells while reducing the impact of anti-inflammatory Treg cells. Additionally, engineered T cells exhibit enhanced performance when LSD1 is inhibited. These findings suggest that LSD1 has an inverse association with T cell function in eliminating tumors, presenting a potential therapeutic target for enhancing cancer immunotherapy.

#### Regulation of B cells

Tumor-infiltrating B lymphocytes (TIBs), present in all stages of cancer, play significant roles in influencing tumor formation and are integral components of the tumor microenvironment. Numerous studies have provided comprehensive insights into the role of epigenetic control in B cell activities [49, 50]. While the role of LSD1 in regulating T lymphocytes is well-defined, its influence on B lymphocyte regulation is less well-understood. However, recent research has indicated that LSD1 plays an essential role in the regulation of B cells, particularly in the development of immunological escape mechanisms. The interaction between LSD1 and the transcription factor B-cell lymphoma 6 (BCL6), which is crucial for defining the germinal center (GC), is vital for controlling B cell function [51]. The development of activated B cells into GC B cells depends on the BCL6. LSD1 deletion can halt BCL6-induced lymphomagenesis. This finding presents a strong rationale for utilizing an LSD1 inhibitor in the treatment of diseases derived from the GC. Notably, inhibitors targeting the protein–protein interaction site of LSD1, rather than its catalytic site, effectively targeted the GC. Another study demonstrated that LSD1 is essential for the proliferation and differentiation of mouse naive B cells into plasmablasts [52]. In the absence of LSD1, cell cycle genes were globally downregulated, which was associated with a decrease in the proliferative capacity of activated B cells. Similar outcomes were observed in the uncontrolled expansion of B cells that contribute to diffuse large B-cell lymphoma (DLBCL), a type of non-Hodgkin lymphoma [53]. LSD1 is found to be overexpressed in human DLBCL tissues, negatively impacting the overall survival rate of DLBCL patients. The selective and potent LSD1 inhibitor ZY0511 was discovered to effectively reduce the growth of DLBCL cells [54]. In DLBCL cells, ZY0511 interacts with LSD1 to increase the methylation of H3K4 and H3K9. Analysis of the transcriptome sequencing data revealed that ZY0511 treatment resulted in a notable enrichment of genes associated with crucial cellular processes such as the cell cycle, autophagy, and apoptosis signaling pathways. ZY0511 prevented cyclin-dependent kinase 4 (CDK4) and cyclin D1 expression, as well as the progression of the G0/G1 cell cycle phase. In DLBCL cells, treatment with ZY0511 dramatically boosted the production of autophagosomes and proteins associated with autophagy. These findings demonstrated that LSD1 regulates B cell activity within the tumor microenvironment through various mechanisms, and inhibiting LSD1 could be beneficial for cancer treatment by promoting immune activation.

#### Regulation of macrophages

There are many solid tumor types in which tumor-associated macrophages (TAMs) play an important role in the TME in various solid tumor types. Several studies have demonstrated the involvement of LSD1 in the infiltration and polarization of TAMs within tumors. In one study, it was described that LSD1 activity leads to the repression of the catalase protein in lipopolysaccharide (LPS) stimulated macrophages, thereby negatively regulating the expression of certain key pro-inflammatory markers [55]. Consequently, the LSD1 inhibitors SP-2509 (which inhibit the LSD1-CoREST interaction) and GSK-LSD1 (which inhibits the FAD binding site of LSD1) have emerged as potential immunosuppressive and anti-inflammatory drugs. These inhibitors have the ability to decrease the transcription of pro-inflammatory cytokines and surface markers associated with M1-macrophage. Phenelzine, however, has been shown to reduce nuclear demethylase activity and increase the transcription and expression of inflammatory M1 macrophage signatures in cellular and triple-negative breast cancer mouse models [56]. Phenelzine is an effective, non-selective and irreversible MAO inhibitor that is commonly used by adults to reduce anxiety and as a treatment for depression. The findings suggest that inhibitors targeting LSD1 should be able to bind to both the FAD and CoREST binding sites to induce or prime macrophages toward M1-like phenotypes. Therefore, single-site targeting LSD1 inhibitors could be potential immunosuppressive drugs capable of limiting M1-macrophage specialization. LSD1 phosphorylation at serine-111 (LSD1-s111p) by chromatin-anchored protein kinase C-theta (PKC-θ), is critical for its demethylase and epithelial-mesenchymal transition (EMT) promoting activity and LSD1-s111p is enriched in chemo-resistant cells in vivo. LSD1 forms a complex with PKC-θ on the epigenetic template of mesenchymal gene, facilitating LSD1-mediated gene induction In experimental models, the combination of chemotherapy with an LSD1 inhibitor enhances the innate immune response mediated by M1 macrophages, resulting in tumoricidal effects [56]. LSD1 inhibition by 2-PCPA reduced the monocyte infiltration in tumors [57]. Besides, LSD1 inhibitors upregulate a set of genes associated with immune response and cytokine-signaling pathways. Additionally, it was observed that the expression of LSD1 decreased during the process of monocyte-to-macrophage differentiation. Mechanistically, LSD1 occupancy at the IL6 promoter decreased, thereby allowing increased H3K4 methylation. Similarly, knocking down LSD1 resulted in an increase in H3K4 methylation at the IL6 promoter and a significant augmentation in the percentage of macrophages [57]. Super-enhancers (SEs) govern macrophage polarization and function. However, the mechanism underlying the signal-dependent latent SEs remodeling in macrophages remains largely undefined. Epigenetic reader zinc finger MYND-type containing 8 (ZMYND8) forms liquid compartments with NF-κB/p65 to silence latent SEs and restrict macrophage-mediated inflammation [58]. LSD1 was discovered to have a crucial functional role within the liquid compartments of ZMYND8, and its recruitment to these compartments is facilitated by ZMYND8 in a manner dependent on p65. The aforementioned studies provide evidence that LSD1 regulates macrophage differentiation and tumor infiltration. In particular, LSD1 inhibition increases the number of M1-like macrophages that possesses the ability to eliminate cancer cells. These findings highlight the potential therapeutic advantages of LSD1 inhibitors in the context of cancer treatment.

#### Regulation of NK cells

NK cells are cytotoxic lymphocytes of the innate immune system that are capable of killing virally infected and/or cancerous cells [59, 60]. They are as effective as T cells but less toxic because they cause fewer immune-related adverse events [61]. Despite the deep understanding of NK cell biology, research on epigenetic regulation of NK cell function is just beginning. NK group 2, member D (NKG2D) is one of the most critical activating receptors expressed by NK cells [62]. There is mounting proof that acute myeloid leukemia (AML) cells can avoid being destroyed by NK cells by expressing little or no NKG2D ligands (NKG2D-Ls) [63]. In the context of low-expressing NKG2D-Ls, it has been observed that CCAAT/enhancer-binding protein a (CEBPA), a highly investigated lineage-specific transcription factor in hematopoiesis, is frequently downregulated or subjected to mutations. Interestingly, the inhibition of LSD1 enzymatic activity using 2-PCPA has been found to have an intriguing effect of restoring the expression of NKG2D-Ls. This restoration is achieved through the induction of CEBPA expression in AML cells. This finding holds promise as a potentially innovative therapeutic strategy for CEBPA-associated AML [64]. Conversely, a different outcome was observed regarding NK cell function when LSD1 was inhibited using a scaffolding inhibitor. The use of scaffolding LSD1 inhibitors demonstrated a strong reduction in the oxidative phosphorylation and glycolysis of NK cells. Moreover, higher doses of these inhibitors induced the generation of mitochondrial reactive oxygen species and led to the depletion of the antioxidant glutathione within the NK cells [65]. The effects described, including the reduction of oxidative phosphorylation and glycolysis, generation of mitochondrial reactive oxygen species, and depletion of the antioxidant glutathione, are specific to scaffolding inhibitors of LSD1 when compared to catalytic inhibitors. Furthermore, these effects are observed primarily in NK cells rather than T-cells. Importantly, the use of scaffolding inhibitors can completely abolish the lytic capacity of NK cells. Similar to the divergent responses of macrophages to LSD1 inhibitors targeting the scaffold site versus the catalytic site, NK cells also exhibit distinct reactions to these different types of inhibitors. Only a few researches have so far examined the relationship between LSD1 and the control of NK cells. In order to better comprehend this mechanism, further descriptive research needs to be investigated.

#### Regulation of DCs

DCs are highly potent antigen-presenting cells that exhibit considerable heterogeneity in terms of cellular phenotypic and functional plasticity. Consequently, there is considerable interest in modulating DCs function to enhance cancer immunotherapy. Various strategies have been developed to target DCs in cancer, including the administration of antigens alongside immunomodulators to mobilize and activate endogenous DCs, as well as the development of DC-based vaccines [66]. In solid tumors, the presence of CD141^Hi^ conventional dendritic cells (CD141^Hi^ cDCs) is essential for effective anti-tumor immunosurveillance and response to immunotherapy. A study demonstrated that patients with myelodysplastic syndromes (MDS) who had lower numbers of CD141^Hi^ cDCs, but not other DC populations, showed reduced overall survival. Interestingly, the pharmacological inhibition of LSD1 facilitated the differentiation of MDS progenitors into CD141^Hi^ cDC. These data suggest that targeting the epigenetic regulation of CD141^Hi^ cDC differentiation offers an intriguing opportunity for intervention and a potential adjunct to immunotherapy for patients with MDS [67].

### Regulation of stromal cells

To facilitate crucial phases in tumor growth, cancer cells recruit supportive cells from the local endogenous tissue stroma [68]. Mesenchymal stromal cells (MSCs), endothelial cells, fibroblasts, stellate cells, and adipocytes are among the stromal cells found in many tumor forms. Once recruited to the TME, these stromal cells release a variety of substances, which regulate angiogenesis, proliferation, invasion, and metastasis.

#### Regulation of MSCs

The stromal microenvironment of tumors, consisting of a mixture of hematopoietic and mesenchymal cells, hamper immune control of tumor growth. However, targeting MSCs with the LSD1 inhibitor tranylcypromine (TCP) has shown promising results. Treating MSCs with TCP induces a stress response involving double-stranded RNA (dsRNA) and its associated elements, including pattern recognition receptors (PRRs), Type-I interferon (IFN1), and IFN-stimulated genes (ISGs). This leads to an overall enhancement in the stability of cell surface peptide: major histocompatibility complexes I (MHC-I) complexes. As a result, TCP-treated MSCs stimulate CD8 T cell activation efficiently and elicit potent anti-tumoral responses [69]. These findings highlight the potential of targeting MSCs to improve immune control and enhance anti-tumor immune responses. Further exploration of these mechanisms may lead to the development of novel therapeutic strategies for combating tumor growth and improving cancer treatment outcomes.

#### Regulation of CAFs

Cancer-associated fibroblasts (CAFs) are a heterogenous group of activated fibroblasts and a prominent component of the tumor stroma, play a significant role in cancer progression. Interestingly, LSD1 expression was found to be elevated in CAFs, acting as a key regulator in the NOTCH3-mediated self-renewal of cancer stem cells (CSCs). In clinical specimens of hepatocellular carcinoma (HCC), the co-occurrence of CAF, LSD1, and NOTCH3 was strongly correlated with unfavorable patient survival outcomes [70]. The association between CAFs, LSD1, and NOTCH3 highlights their potential as therapeutic targets for HCC and underscores the importance of understanding the stromal contribution to cancer progression for the development of effective treatment strategies. Hence, inhibiting LSD1 could potentially suppress the activity of CAFs and mitigate their immunosuppressive effects. Additionally, studies have revealed increased expression of LSD1in lung tissues from mice with bleomycin-induced pulmonary fibrosis, as well as lung fibroblasts treated with transforming growth factor-β1 (TGF-β1) [71]. LSD1 knockdown was found to impede the differentiation of fibroblasts into myofibroblasts, thereby inhibiting the development of pulmonary fibrosis. Suppression of TGF-β1/ SMAD family member 3 (SMAD3) signaling pathway through histone H3K9 methylation and histone H3K4 methylation upregulation responsible for the fibroblast differentiation inhibition in LSD1 inactivated cells. Myofibroblasts are a subtype of cancer-associated fibroblasts that are in charge of tumor cells' immune resistance [72]. Following mono chemotherapy, the CAF markers fibroblast-activation protein (FAP) and C–C motif chemokine ligand 2 (CCL2) have been reported to increase in the tumor microenvironment. However, these markers are decreased when LSD1 is inhibited, either as a standalone treatment or in combination with chemotherapy [56]. Based on these discoveries, inhibiting LSD1 can potentially reduce the activation of CAFs and enhance the immune system’s capacity to eliminate cancer cells. However, further comprehensive research is required to fully understand the underlying molecular mechanism involved in this process.

#### Regulation of ECs

The endothelium, which is a thin monolayer made up of endothelial cells (ECs), is able to direct the development and proliferation of the connective tissue cells that make up the blood vessel wall's layers. The crucial process of the development of new blood vessels is controlled by the interaction between tumor cells and ECs [73]. There is mounting evidence that suggests alterations in ECs play a role in the development of cancer [74]. In order to decrease the antitumor immune response and/or activate receptors on tumor cells, tumor endothelial cells release cytokines, which attenuate the immune cells’ cytotoxic reactions [75]. Martyna Wojtala and colleagues conducted a study to investigate the role of LSD1 in modulating the inflammatory state of ECs. They utilized two approaches to analyze this function: the use of the LSD1 inhibitor 2-PCPA and the knockdown of LSD1 through short hairpin RNA (shRNA) in a cell model. The results of the study demonstrated that inhibiting LSD1 activity led to a decrease in the secretion of proinflammatory cytokines, namely IL6 and IL8. Moreover, the researchers observed a reduction in the secretion of chemokines CCL2, CCL5, and C-X-C motif chemokine 10 (CXCL10), which play crucial roles in the recruitment of leukocytes to sites of inflammation. Overall, the inhibition of LSD1 was found to downregulate the proinflammatory actions of ECs [76]. Another research group employing a similar approach reported that inhibiting LSD1 in endothelial cells resulted in abnormal cell aggregation during the G2/M phase of the cell cycle, displaying distinctive characteristics of pulverization. This included chromosomal breaks and gaps (≤ 20), lost and lagging chromosomes, acentric fragments, segregation defects, and chromosome bridges. The upregulation of checkpoint kinase 1 (CHK1) expression and phosphorylation was observed as a consequence of LSD1 inhibition. Additionally, an increase in DNA damage was observed, accompanied by the activation of ATR/ATR-IP signaling indicated by serine 139 phosphorylation of H2AX [77]. The findings of decreased inflammatory function and aberrant cell proliferation upon LSD1 inactivation highlight the essential role of LSD1 in the functioning of ECs. However, the specific mechanisms by which LSD1-mediated regulation of ECs controls immune responses in tumors have not been investigated thus far.

#### Regulation of adipocytes

Adipocytes, which are a key type of stromal cells found in several organs, are believed to play an active role in the tumor microenvironment. These specialized fat cells, known as cancer-associated adipocytes (CAAs), not only reside in close proximity to cancer cells but also engage in interactions by releasing various substances that can exert both local and systemic effects [78]. Tumor cells induce the transformation of normal adipocytes into CAAs, which function as metabolic parasites that can be recognized by their engulfment of metabolites from the stroma. Although the direct relationship between LSD1 and CAAs has not been examined yet, various research groups have investigated the impact of LSD1 on typical adipocytes. These studies have uncovered that LSD1 stimulates oxidative metabolism and facilitates the conversion of white adipose tissue (WAT) into brown adipocytes. During this process, LSD1 interacts with nuclear respiratory factor 1 (NRF1), resulting in the activation of genes related to mitochondrial biogenesis and oxidative phosphorylation [79]. LSD1 inhibitors reduce the differentiation of brown adipocytes, and Similar outcomes are observed by RNAi-mediated LSD1 knockdown, which can be reversed by expressing wild-type LSD1 but not catalytically inactive LSD1. Mechanistically, LSD1 works to promote brown adipogenesis by inhibiting the Wnt signaling pathway [80]. In aging inguinal white adipose tissue, the levels of LSD1 decrease simultaneously with the decline of beige fat cells, a subtype of adipose cells known for their ability to generate heat and burn energy [81]. It has been discovered that beige adipocytes promote cancer growth [82]. By regulating the expression of the peroxisome proliferator-activated receptor α (PPAR α), LSD1 plays a role in maintaining normal levels of beige adipocytes. Loss of beige adipocytes occurs as a result of LSD1 ablation, however, this loss can be recovered with the help of a Pparα agonist [83]. In conclusion, the findings indicate that LSD1 regulates the adipocytes that promote tumor growth. This suggests that targeting LSD1 could hold therapeutic potential in reducing tumor-promoting adipocytes. By specifically inhibiting or modulating the activity of LSD1, it may be possible to intervene in the interaction between adipocytes and cancer cells within the tumor microenvironment. Further research and clinical studies are needed to explore the therapeutic potential of LSD1 inhibitors or modulators in the context of adipocyte-mediated tumor progression.

#### Regulation of stellate cells

Stellate cells, which were first discovered in the liver's perisinusoidal regions, facilitate wound healing through the release of growth factors and extracellular matrix. The significance of these cells in the development and spread of tumors has received more attention in recent years. Numerous epigenetic processes, including DNA methylation, histone modification, and the development of certain chromatin structures, play significant roles in the gene transcriptional expression in stellate cells that control many essential functions. Following prolonged liver injury, hepatic stellate cells (HSCs) undergo a process of differentiation and transform into myofibroblasts, which play a crucial role in the development of liver fibrosis. In LSD1 knockdown HSCs and HSCs treated with the LSD1 inhibitor HCI-2509, the levels of histone deacetylase 3 (HDAC3), enhancer of zeste homolog 2 (EZH2), and methyl CpG binding protein 2 (MeCP2) were found to be reduced. These proteins have previously been shown to be associated with fibrotic characteristics. Moreover, the inhibition of LSD1 leads to modifications in the gene and microRNA expression patterns within HSCs. Consequently, the fibrogenic markers collagen I and smooth muscle actin (SMA) exhibit decreased expression levels, while PPAR gamma (PPARγ) shows enhanced expression upon LSD1 silencing [84].

### Regulation of cytokine

**Fig. 3:**
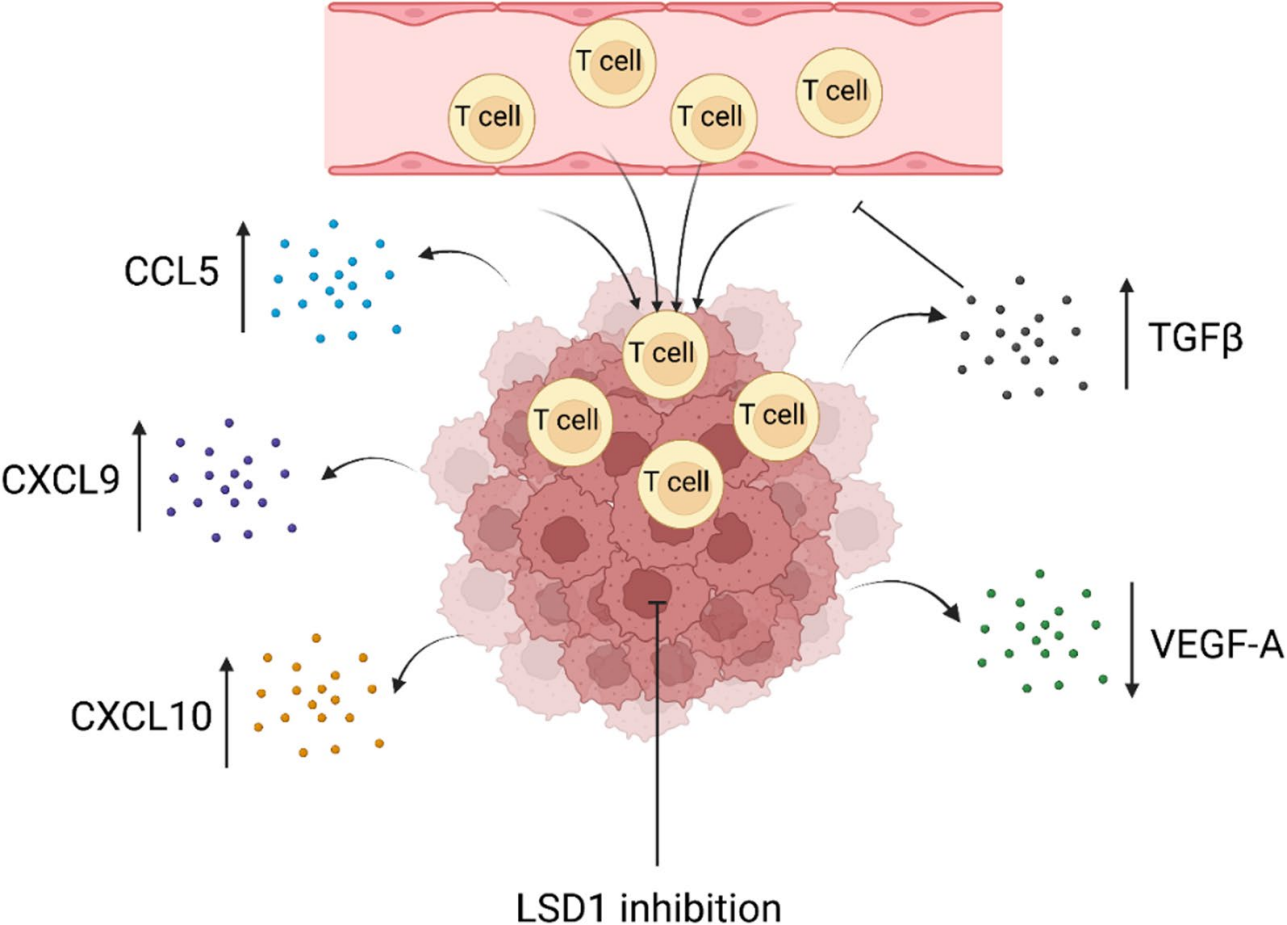
LSD1 inhibition results in CD8 T cell attracting chemokines secretion. However, an increased amount of TGFβ secretion may create obstacles in this process, which can be overcome by TGFβ inhibition. “Figure created with BioRender.com”

Cytokines are major regulators of the TME, enabling communication between immune system cells over short distances [85]. A group of cytokines, known as chemokines, is responsible for immune cell infiltration in tumor sites [86]. LSD1 expression or its inactivation modulates various functions of cytokines or chemokines (Fig. 3). In silico analysis of TCGA data reveals that the expression of LSD1 is inversely associated with the levels of cytotoxic T cell-attracting chemokines, such as CCL5, CXCL9, CXCL10 in clinical triple-negative breast cancer (TNBC) specimens [87]. A supporting study demonstrated that using LSD1 inhibitors or depletion of LSD1 by siRNA significantly increased the expression of CCL5, CXCL9, and CXCL10. Conversely, overexpression of LSD1 attenuated the expression of these genes. Furthermore, treatment with siRNA or inhibitor of chemokine receptors blocked LSD1 inhibitor-enhanced CD8 T cell migration, indicating a critical role of chemokines in LSD1-mediated CD8 T lymphocyte trafficking to the tumor microenvironment [87]. Interestingly, it was noted that either depletion or overexpression of LSD1 exerted negligible effects on the expression of other types of chemokines such as CCL2, CCL3, or CCL4 whose activities are known to have pro-tumor roles, suggesting that targeting LSD1 may have a favorable impact on promoting antitumor immunity [88]. The TGFβ is a crucial cytokine that is hijacked by the tumor to increase fibrotic stroma, promote epithelial to mesenchymal transition, promote metastasis, and create an immune-suppressed microenvironment that shields the tumor from recognition by the immune system [89]. LSD1 is an integral component of the nucleosome remodeling and deacetylase (NuRD) complex [6]. Analysis of transcriptional targets has revealed that LSD1/NuRD complex regulates several cellular signaling pathways including the TGFβ signaling pathway [43]. LSD1 is downregulated in breast carcinomas and its level of expression is negatively correlated with that of TGFβ. Another study also reported similar findings, demonstrating that LSD1 ablation strongly induced the TGFβ family members (TGFβ1, TGFβ2 and TGFβ3). This upregulation was largely suppressed when LSD1 was re-introduced into the cells [25]. However, the opposite scenario has been seen in non-small-cell lung carcinoma (NSCLC). Where it was found that a protein SEPTIN 6 (SEPT6) was stabilized by LSD1 which facilitates liver fibrosis partly through the TGFβ1/SMAD pathway [90]. Downregulated LSD1 could prevent pulmonary fibrosis by suppressing the TGFβ1/SMAD3 pathway, which is achieved by regulating the balance between H3K4 or H3K9 methylation. Elevated expression of LSD1 was found to correlate with prostate cancer recurrence and with increased vascular endothelial growth factor A (VEGFA) expression [91]. Functional depletion of LSD1 expression using siRNA in prostate cancer cells decreases VEGFA and blocks androgen-induced VEGFA, prostate-specific antigen (PSA) and transmembrane serine protease 2 (TMPRSS2) expression and reduces proliferation of both androgen-dependent and independent prostate cancer cells.

#### Significance of targeting LSD1 for the improvement of immune checkpoint therapy

**Fig. 4:**
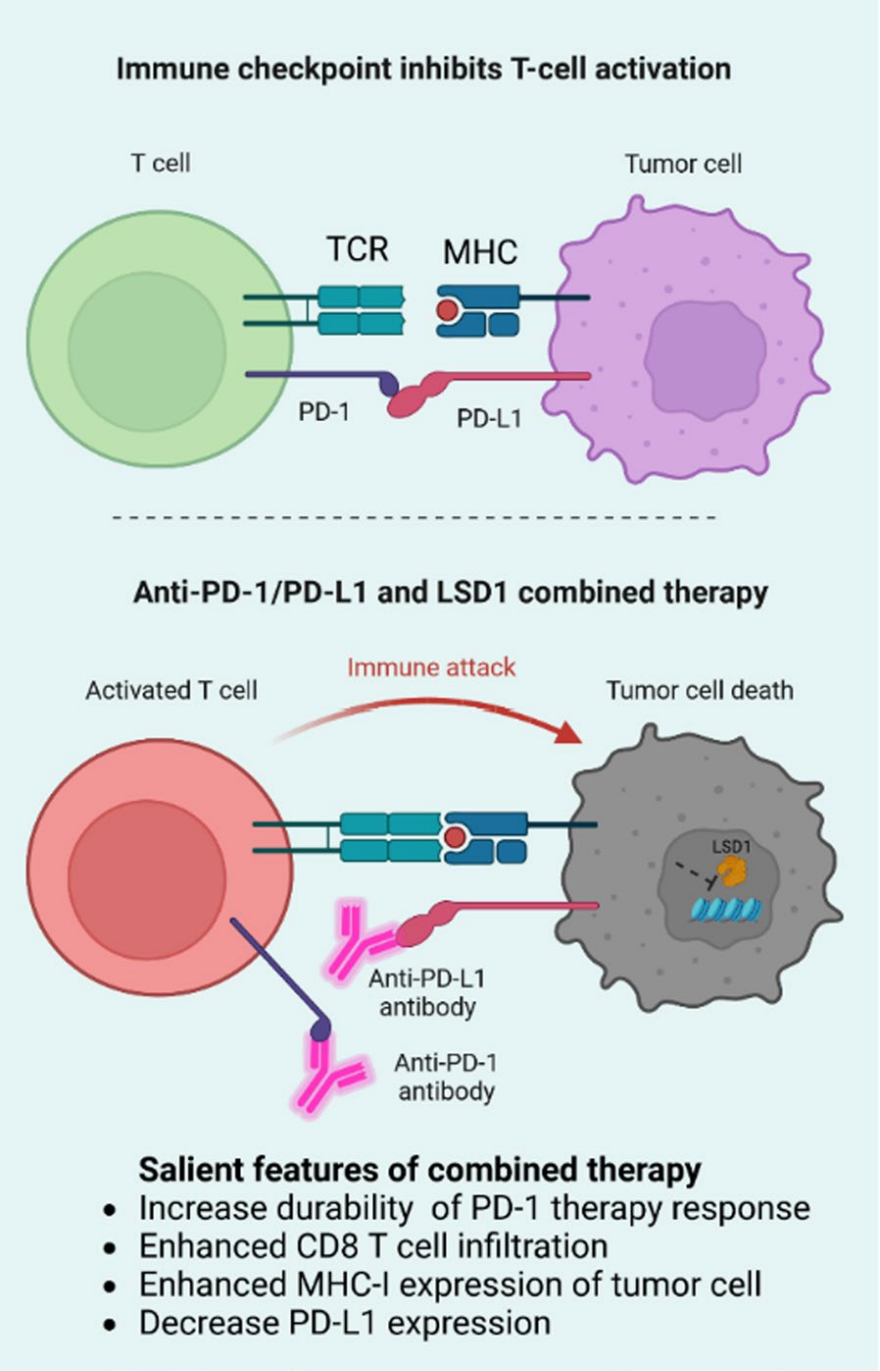
Picture represents the combined effect of LSD1 inhibitor and ICB therapy on CD8 T cell action. “Figure created with BioRender.com”

Blocking immune checkpoints is considered one of the most promising approaches to elicit therapeutic antitumor immune responses. The forefront of immunotherapy for various types of malignancies involves inhibiting immune regulatory checkpoints, notably CTLA-4 and the PD-1-PD-L1 axis [92]. Other immunological checkpoints that are being considered as targets include Lymphocyte-activation gene 3 (LAG3), T cell immunoglobulin and mucin domain-containing protein 3 (TIM3), and T cell immunoglobulin and ITIM domain (TIGIT) [92]. The field of cancer treatment has been revolutionized by the development and clinical utilization of immune-checkpoint (IC) inhibitors that specifically target CTLA4, PD-1, and PD-L1. However, it is noteworthy that a majority of patients fail to experience clinical benefits when these therapies are used alone or in combination [93]. Therefore, it is crucial to continue discovering drugs that target additional immunological checkpoints, co-stimulatory receptors, and/or co-inhibitory receptors that regulate T-cell function. Here, we described the effect of LSD1 in combination with ICB or alone to modulate the tumor-killing capacity of CD8 T cells (Table 1, Fig. 4).

**Table 1:** LSD1 in combination with immune checkpoint inhibition

IC protein	Treatment type	Treatment effects	Type of cancer	References
PD-1	Combination of LSD1 and PD-1 inhibition	Increase the longevity of tumor-killing capacity of CD8 T cells	Colon cancer	[43]
	LSD1 inhibition	Increase PD-1 mRNA and protein	Infiltrated CD8 T cell in a mouse melanoma model	[38]
	Combination of LSD1 and PD-1 inhibition	1) Induces cytotoxic CD8 T cell infiltration 2) Enhance in vivo breast tumor immunogenicity 3) Inhibit metastasis	Breast cancer	[87]
	Combination of LSD1, TGFβ and PD-1 blockade	Increase infiltration and cytotoxicity of CD8 T cells	Melanoma	[25]
PD-L1	LSD1 inhibition	1) Increase effectiveness of CD8 T cell-mediated cytotoxicity 2) Decrease PD-L1 expression	Hepatocellular carcinoma	[97]
	LSD1 inhibition	Decrease exosomal PD-L1 and restore CD8 T cell response	Gastric cancer	[41]
	LSD1 inhibitor combined with PD-L1 antibody	Decrease PD-L1 expression	Cervical cancer	[37]
	LSD1 deletion	Increase PD-L1 expression	Head and neck squamous cell carcinoma	[98]
CTLA4	Combination of LSD1, PD-L1 and CTLA4 inhibition in SWI/SNF mutatated cells	Increase PBMC penetration	Ovarian clear cell carcinomas and small cell carcinoma of the ovary hypercalcemic type	[99]

#### Effect on PD-1 regulation

As we have stated, PD-1/PD-L1 blocking therapy is a promising strategy that has transformed the anticancer treatment landscape. Despite this, resistance to the use of PD-1/PD-L1 blockade continues to pose significant challenges to its wider application. Considerable effort has been made to overcome these limitations, including the use of combination therapies [94]. When there are genetic alterations or when small molecules specifically targeting LSD1 are used in conjunction with anti-PD-1, the progenitor-exhausted CD8 T cells are preserved. This preservation ensures a continuous supply of cells that have the ability to multiply and effectively combat tumors, which in turn enhances the lasting effectiveness of anti-PD-1 therapy [43]. Moreover, there are dynamic changes in H3K4 at the PD-1 site during the activation of CD8 T cells, indicating that LSD1 might be essential in managing PD-1 expression [38]. It was observed that CD8 T cells lacking LSD1, which infiltrated the tumors, showed a higher expression of PD-1 compared to the normal CD8 T cells. The study further revealed that B-lymphocyte-induced maturation protein-1 (BLIMP-1) brings LSD1 to the PD-1 site, which then helps in eliminating the histone marks that activate and control PD-1 expression. Interestingly, there was no apparent therapeutic effect was observed when using the anti-PD-1 antibody alone in mice bearing xenografts of TNBC tumors. However, when combining LSD1 inhibitors with PD-1 antibodies, there was a reduction in tumor growth and pulmonary metastasis, along with decreased Ki-67 levels and enhanced CD8 T cell infiltration [87]. However, the reduction of LSD1 in several tumor cells induces TGFβ expression, which constrains the T-cell immune action through suppressing intratumoral CD8 T-cell cytotoxicity, which minimizes the antitumor effects of LSD1 inhibition-induced T-cell infiltration [25]. Consequently, infiltration and cytotoxicity of CD8 T cells are significantly increased when LSD1 and TGFβ are simultaneously depleted and PD-1 blocked [25]. The phosphorylation at serine 111 of nuclear LSD1 (nLSD1p) is essential for the development of breast cancer stem cells. As compared to traditional FAD-specific LSD1 catalytic inhibitors, selective LSD1 inhibitors such as GSK2879552 better inhibit the stem-like mesenchymal signature [95]. It has been found that PD-1^+^CD8^+^ T cells from resistant melanoma patients and 4T1 immunotherapy resistant mice are enriched in nLSD1p. Combining immunotherapy with selective targeting of the LSD1p nuclear axis enhances CD8 T cell infiltration into tumors of 4T1 immunotherapy-resistant mice.

#### Effect on PD-L1 regulation

Current biomarkers of anti-PD-1 or anti-PD-L1 antibody selection widely rely on the PD-L1, which is widely validated, used and accepted globally [96]. Indeed, the relationship between LSD1 inhibition and PD-L1 expression appears to be context-dependent and can vary among different cancer types. While LSD1 inhibition has been reported to upregulate PD-L1 expression in certain cancer cells, it has also been observed to downregulate PD-L1 expression in others. MiR-329-3p decreases the expression of LSD1 mRNA in hepatocellular carcinoma, leading to the suppression of immunosuppressive properties in tumor cells and increased effectiveness of T cell-mediated cytotoxicity. Research has indicated that when myocyte enhancer factor 2D (MEF2D) is demethylated, it can attach itself to the PD-L1 promoter and activate its expression. In this process, LSD1 plays a key role in demethylation. When LSD1 is inactivated, the ability of MEF2D to activate PD-L1 is diminished [97]. A significant study showed that LSD1 KO in mouse forestomach carcinoma (MFC) cells resulted in significantly slower cell growth in immunocompetent 615 mice compared to immunodeficient BALB/c mice. The studies also observed that PD-L1 accumulation in exosomes of gastric cancer cells inhibited the antitumor response of T-cells, while membrane PD-L1 remained constant in LSD1 KO cells. These results indicate that LSD1 inhibits the T-cell response in cancer cells by utilizing exosomes as vehicles for PD-L1, while the deletion of LSD1 restores T-cell response [41]. In cervical cancer, when LSD1 was knocked down, PD-L1 expression was directly downregulated through increased levels of H3K4me2 in PD-L1 promoters [37]. Here, by targeting the 3’ untranslated regions (3'UTRs) of PD-L1, the LSD1/wild-type p53/miR-34a signaling axis regulates PD-L1 expression. Consequently, researchers found that combining an LSD1 inhibitor (ORY-1001) with anti-PD-L1 monoclonal antibodies effectively inhibited tumor growth in established subcutaneous xenograft models [37]. In order to preserve the characteristics of cancer stem cells (CSCs), LSD1 expression controls the expression of BMI-1. Tumor LSD1 ablation inhibits tumorigenicity in immune-deficient xenografts in vitro and suppresses CSC-like characteristics in vivo [98]. This deletion, however, upregulates PD-L1 levels in a mouse model with immune competence, compromising antitumor immunity and reducing antitumor effectiveness. However, the combination of LSD1 inhibitors and anti-PD-1 monoclonal antibodies overcomes tumor immune evasion mediated by PD-L1 upregulation and markedly inhibits tumor growth in immunocompetent tumor-bearing mouse models. In a 3D immune-organoid platform, LSD1 inhibitor, SP-2577 stimulated IFN-dependent antitumor immunity and PD-L1 expression in small-cell carcinoma cells [99]. The information mentioned above indicates that inhibiting LSD1 can lead to decreased PD-L1 expression in certain types of cancer, while in others it can cause an increase. Nonetheless, in both scenarios, employing a combination of LSD1 inhibitors with immune checkpoint therapy has been observed to reduce tumor size and enhance the ability of CD8 T cells to kill cancer cells.

#### Effect on CTLA-4 regulation

Another IC protein, CTLA-4 is mainly expressed on the surface of activated T lymphocytes. It has a close relationship with the costimulatory molecule receptor (CD28) on the surface of T cells in terms of gene structure, chromosome localization, sequence homology and gene expression [100–102]. The regulatory relationship between LSD1 and CTLA-4 has not been explored, but a study has reported that the combination of LSD1 inhibitor (SP-2577), α-PD-L1, and α-CTLA-4 treatment can significantly enhance the penetration of PBMC in ovarian cancer with SWI/SNF mutation [99]. This suggests that inhibition of LSD1 has some unknown regulatory mechanism on the immune checkpoint CTLA-4.

In summary, the potential of inhibiting LSD1 as a supplementary treatment in combination with immunotherapy presents a promising and innovative approach in the development of cancer therapies. The limited research conducted so far on the impact of LSD1 inhibition on few IC proteins warrants further investigation to better understand its effects and implications. A more comprehensive examination of this relationship could provide valuable insights and aid in the development of more effective and targeted cancer treatments. By better understanding the interplay between LSD1 inhibition and immunotherapy, researchers and medical professionals may be able to devise new therapeutic strategies with improved outcomes for cancer patients.

### Role of LSD1 in antigen presentation

T cells do not directly recognize antigens; instead, they recognize antigens that are presented by major histocompatibility complexes (MHCs) using their TCR [103]. MHC class I (MHC-I) molecules are responsible for binding peptides produced from a cell’s genes and then carrying and presenting this antigenic data on the cell surface. By doing so, CD8 T cells can recognize and target pathogenic cells that are producing abnormal proteins, such as cancer cells expressing mutated proteins. In a syngeneic model of small cell lung cancer (SCLC), LSD1 inhibitor bomedemstat substantially enhanced CD8 T cell infiltration and inhibited tumor growth. Additionally, bomedemstat increased MHC-I expression in mouse SCLC tumor cells in vivo. As a result, tumor-specific T cells were more likely to kill tumor cells in cell culture stimulated by bomedemstat [104]. By specifically inhibiting LSD1, there is a restoration of MHC-I expression along with the transcriptional activation of genes associated with antigen presentation. Furthermore, combining LSD1 inhibitors with ICB enhances antitumor immunity in refractory SCLC models. Collectively, the combination of LSD1 inhibition with ICB has been demonstrated to improve therapeutic response in SCLC by modulating MHC-I antigen presentation [40]. In studies using organoids, functional interactions between murine or patient-derived mammary tumor organoids and tumor-specific cytotoxic T cells were examined through high-throughput screening. It was found that the LSD1 inhibitor (GSK-LSD1) exhibited antitumor effects and increased MHC-I-mediated antigen presentation in a mouse mammary tumor model [105]. When MSCs are treated with another LSD1 inhibitor TC, it induces intracellular dsRNA stress. This stress leads to the production of IFNβ and enhances the expression and stability of MHC-I molecules on the cell surface. As a result, TC-treated MSCs can effectively present immunogenic peptides to CD8 T cells, thereby stimulating a robust anti-tumor immune response [69]. In addition to its other effects, LSD1 has been observed to impact MHC class II molecules (MHC-II). When macrophages are treated with Phenelzine, an LSD1 inhibitor, there is an increase in the expression of certain MHC-II genes. This, in turn, enhances the ability of MHC-II molecules to present antigens to T cells [106]. In summary, the depletion of LSD1 has the potential to boost the activity of antigen-presenting molecules. This enhancement in activity increases immunogenicity and reinvigorates the response of CD8 T cells to anti-tumor therapies.

### LSD1 inhibitors with immunologic activity

**Fig. 5:**
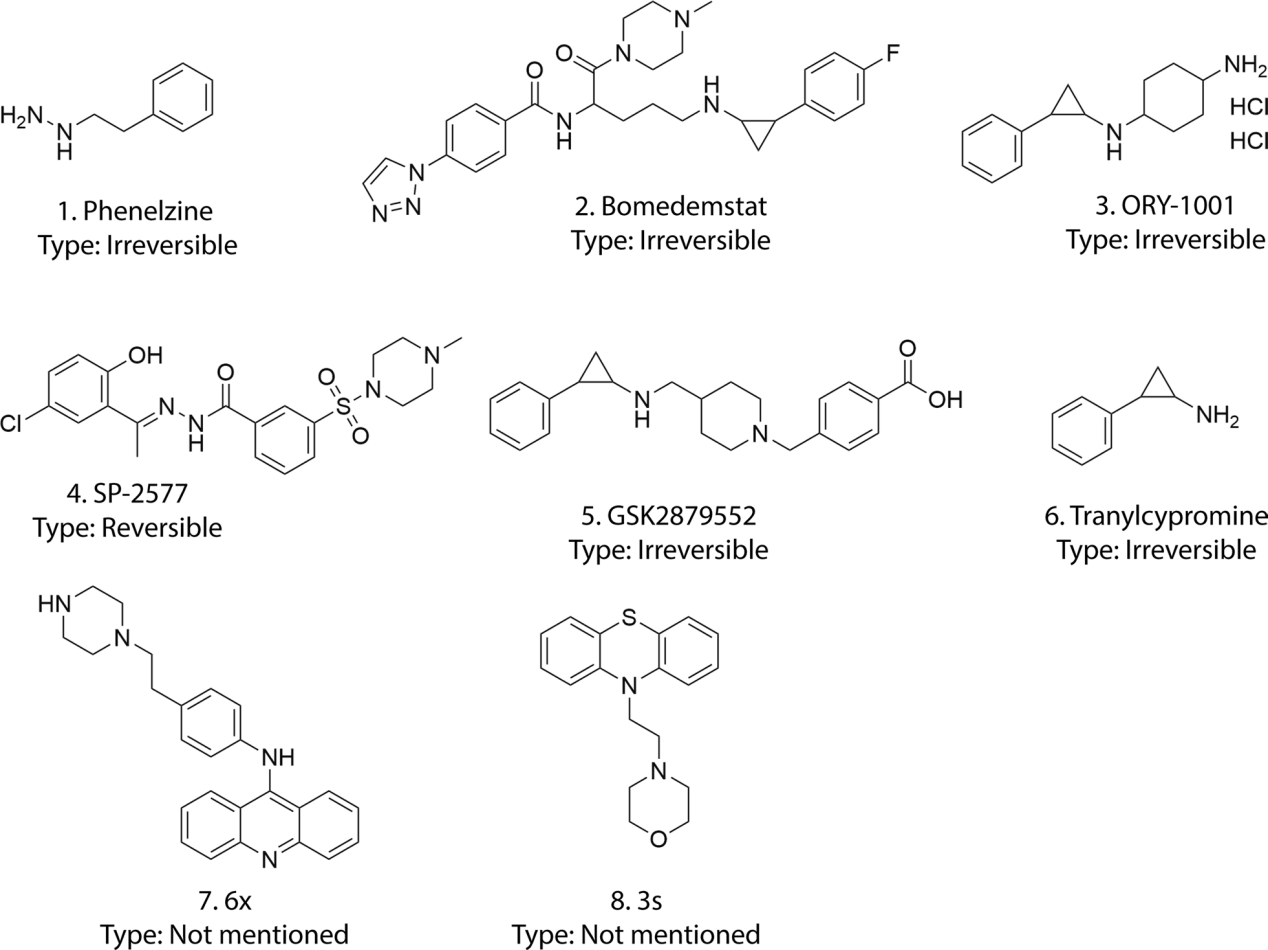
The figure represents LSD1 inhibitors containing the immunomodulating ability

Although a significant number of LSD1 inhibitors have been reported to date in various studies, only a few inhibitors have been studied in terms of immune regulation (Table 2, Fig. 5) [107]. LSD1 inhibitors have shown varying effects on different types of immune cells. For instance, phenelzine treatment (which inhibits both the catalytic and scaffolding function of LSD1) has been found to activate the inflammatory M1-like subtype of macrophages. On the other hand, inhibiting the demethylase function of LSD1 using GSK-LSD1 or inhibiting its interaction with CoREST using SP-2509 has been shown to activate immunosuppressive M2-like macrophage properties [55, 56, 106]. In recent years, phenelzine, an authorized MAO inhibitor that is generally used for psychiatric purposes, has also been recognized as an inhibitor of LSD1 [108]. In patient-derived circulating tumor cells (CTCs), combined chemotherapy and phenelzine treatment eliminated the mesenchymal signature and decreased the stem-like signature. Macrophages treated with phenelzine exhibited gene expression patterns consistent with the M1 phenotype, which is typically induced by IFN-γ and LPS [106]. In vivo study indicates that Inhibition of LSD1 by phenelzine helps reduce the negative effects of chemotherapy, including the processes of EMT and CAFs infiltration, while promoting the infiltration of M1 macrophages. A second small molecule targeting LSD1 called bomedemstat (IMG-7289), is currently being evaluated in phase 1 clinical trials. In numerous preclinical investigations, bomedemstat demonstrates anti-cancer effects, which include slowing the proliferation of cancer cells, inducing apoptosis, and enhancing T-cell-mediated tumor death [12, 39, 109]. The outcomes from a study utilizing an immunocompetent syngeneic mouse model suggest that by employing the bomedemstat for epigenetic reprogramming of SCLC cells, it is possible to counteract the inherent immunosuppressive mechanisms in SCLC, such as MHC-I downregulation. This process can subsequently increase the sensitivity of SCLC tumors to immune detection and destruction [104]. ORY-1001, a clinically utilized LSD1 inhibitor, has been employed as a medication to treat acute leukemia [110]. There have been limited investigations into the immunomodulatory effects of ORY-1001. A study revealed that combining ORY-1001 with anti-mouse CD47 or PD-L1 monoclonal antibodies resulted in more potent inhibition of tumor growth compared to using either drug alone [37]. In a study by Yihui Zhai et al. ORY-1001 was administered using the PD-1-engineered epigenetic nanoinducer OPEN. It was observed that ORY-1001 upregulated intratumor interferons (IFNs) and downstream MHC-I and PD-L1 expressions. Additionally, OPEN effectively inhibited the production of PD-L1 induced by IFN. The intratumoral concentrations of overall and functional cytotoxic T cells were increased 8- and 29-fold, respectively, and the growth of transplanted tumors was severely inhibited [111]. SP-2577 has shown increased levels of PD-L1 in ovarian cancer cases associated with mutations in the Switch/Sucrose-Nonfermentable (SWI/SNF) complex. As a result, co-treatment with α-PD-L1 antibodies greatly enhanced the infiltration of CD8 T cells [99]. In NK cells, SP-2577 eliminates the lytic capacity and potently decreases oxidative phosphorylation and glycolysis. Glutathione supplementation can, however, restore NK cell cytolytic activity [65]. GSK2879552, an irreversible LSD1 inhibitor, has exhibited a durable PD-1 blocking response and a synergistic effect in the regulation of tumor growth [43]. By inhibiting LSD1, GSK2879552 enhances the immune system’s ability to block PD-1 signaling, thus allowing immune cells to effectively recognize and attack tumor cells. TCP treatment was found to enhance the stability of MHC-I complexes on the cell surface, leading to the activation of CD8 T cells and the generation of potent antitumor responses [69]. Recently, a study by Hui-Min Liu et al. discovered an amsacrine-based LSD1 inhibitor 6x, which increased the ability of T cells to eradicate tumors in mouse xenograft tumor model [112]. Mechanistic research showed that compound 6 × hindered the stemness and movement of gastric cancer cells while reducing the expression of PD-L1. Another recent study found that the antipsychotic medication chlorpromazine is an LSD1 inhibitor [113]. Researchers synthesized a series of chlorpromazine derivatives, with compound 3 s being the most effective. Crucially, compound 3 s not only inhibited LSD1 at a cellular level but also reduced the expression of PD-L1 in gastric cancer cells, ultimately improving the T-cell killing response in in vivo study. In conclusion, when LSD1 inhibitors are combined with immune checkpoint blockade (ICB) therapy, there is a potential to enhance CD8 T cell infiltration and function within the tumor TME. Additionally, LSD1 inhibitors might affect the expression of immune checkpoint molecules, including PD-L1, on tumor cells or immune cells within the TME. This modulation can synergize with ICB therapy, leading to enhanced blockade of immune checkpoints and unleashing the full potential of CD8 T cell-mediated antitumor immunity. Further studies are required to investigate the intricate interplay between LSD1 inhibition, immune cell activation, immune checkpoint modulation, and other components of the TME.

**Table 2:** LSD1 inhibitors’ effects on immune functions

SI	Name	Category	Immunologic activity	Ref
1	Phenelzine	Irreversible MAO inhibitor	• Inhibit FAD and CoREST binding to LSD1 • Increase transcription and activation of M1 macrophage signature genes • Decrease CAF-mediated resistance to chemotherapy	[56]
2	Bomedemstat	Irreversible LSD1 inhibitor	• Increase MHC-I expression • Increase CD8 T cell infiltration in tumor	[104]
3	ORY-1001	Irreversible FAD binding LSD1 inhibitor	• Combined with PD-L1 antibody therapy it increases tumor cell killing	[37]
4	SP-2577	Reversible LSD1 inhibitor	• Stimulate IFN-dependent antitumor immunity	[99]
5	GSK2879552	Irreversible LSD1 inhibitor	• Prolong responses to PD-1 blockade	[43]
6	Tranylcypromine	Irreversible LSD1 inhibitor	• Increase MHC-I stabilization • Stimulate CD8 T cell activation	[69]
7	Amsacrine derivative compound 6x	LSD1 inhibitor	• Suppress expression of PD-L1 • Promote T-cell killing response	[112]
8	Chlorpromazine derivative compound 3 s	LSD1 inhibitor	• Suppress expression of PD-L1 • Promote T-cell killing response	[113]

## Conclusion and perspectives

LSD1 is an exciting target for improving immunotherapy, as it plays a crucial role in regulating anti-tumor activity within the TME through newly discovered mechanisms. The methylation of H3K4 in cells and the regulation of gene expression in the immune microenvironment are closely interconnected. These studies have demonstrated that inhibiting LSD1 enhances the antitumor effects of TME through various mechanisms. Firstly, LSD1 inhibition has been shown to increase CD8 T cell infiltration into the tumor, thereby promoting cytotoxicity and tumor cell killing. Additionally, it inhibits the immunosuppressive actions of regulatory T cells, allowing for a more robust immune response against the tumor. Furthermore, LSD1 inhibition enhances natural killer (NK) cell cytotoxicity, further contributing to the antitumor effect. Moreover, LSD1 inhibition has been implicated in increasing the durable response of CD8 T cells. This suggests that it may reduce T cell exhaustion, a state in which T cells lose their effectiveness, proliferative capacity, and express inhibitory immune checkpoint receptors. Epigenetic remodeling plays a significant role in preventing T cell reinvigoration when immune checkpoints are blocked and is crucial for T cell-mediated tumor-killing activity [114].

Although a few significant studies demonstrated the relationship between LSD1 and TME, many questions in the field remain unanswered. While the effects of LSD1 on several immune cells have been studied, there is still a lack of in-depth exploration of other immune cells such as B cells, neutrophils, and more. A study involving zebrafish has described a regulatory network involving Gfi1aa, LSD1 and CEBPA that controls neutrophil development [115]. However, its relation to cancer has not been studied yet. Besides, the effect of LSD1 inhibition on other immune checkpoints such as CTLA-4, LAG3, TIM3, and TIGIT requires further exploration. We have seen that the LSD1 inhibitor containing both FAD and scaffold site inhibition is more effective in modulating the TME compared to a single-target inhibitor focusing solely on FAD or scaffold. This suggests that improving dual-site targeting LSD1 inhibitors could serve as potent immunomodulating agents for future anticancer therapy. Besides, LSD1 inhibitors have demonstrated an increase in M1-macrophage infiltration and T cell-mediated tumor destruction, as well as a reduction in the immunosuppressive functions of CAFs. Additionally, recent studies involving amsacrine and chlorpromazine derivatives have shown increased T cell responses in in vivo experiments, indicating a need to continue enhancing these inhibitors for more effective cancer treatments. The concept of combining epigenetic therapy with ICB therapy presents a novel approach to cancer treatment, particularly considering the ongoing need to improve ICB therapy and the growing problem of resistance to different ICB therapies. Therefore, LSD1 inhibition in combination with ICB therapy holds promise as an effective strategy for future cancer therapy.

## Data Availability

Not applicable.
